# Biodistribution of free Francium-221 and Bismuth-213 in Tumour-bearing SCID mice after successful development of Actinium-225/Francium-221 radionuclide generator Set-up

**DOI:** 10.1007/s00259-025-07427-4

**Published:** 2025-07-01

**Authors:** Sabine Zitzmann-Kolbe, Yvonne Remde, Ingrid Moen, Balázs Madas, László Mázik, Frans Suurs, Steffen Happel, Martin Schäfer, Christoph Schatz, Harun Taş, Urs B. Hagemann, Martina Benešová-Schäfer

**Affiliations:** 1 Bayer AG, Berlin, Germany; 2 Service Unit for Radiopharmaceuticals and Preclinical Studies, German Cancer Research Center (DKFZ), Im Neuenheimer Feld 280, Heidelberg, 69120 Germany; 3 Bayer AS, Oslo, Norway; 4 Environmental Physics Department, Institute for Energy Security and Environmental Safety, HUN-REN Centre for Energy Research, Konkoly-Thege Miklós út 29-33, Budapest, 1121 Hungary; 5 Doctoral School of Physics, ELTE Eötvös Loránd University, Pázmány Péter sétány 1/A, Budapest, 1117 Hungary; 6 TrisKem International, 3 rue des Champs Geons, Bruz, 35170 France; 7 Research Group Translational Radiotheranostics, German Cancer Research Center (DKFZ), Im Neuenheimer Feld 280, Heidelberg, 69120 Germany; 8 Life Molecular Imaging, Berlin, Germany

**Keywords:** Targeted alpha therapies, Actinium-225, Francium-221, Bismuth-213, Radionuclide generators, Radionuclide-specific organ accumulation

## Abstract

**Purpose:**

Despite the clinical evidence of actinium-225 (^225^Ac)-based targeted alpha therapies (TαT) efficacy, optimized treatment regimens are needed to improve overall clinical response rates and decrease toxicities. The nuclear recoil effect of ^225^Ac and its resulting daughter nuclides have been hypothesized to contribute to non-targeted damage. However, a lack of generator concepts for radionuclidically pure francium-221 (^221^Fr), involvement of strong acids for elution, and its short half-life (4.8 min), has limited in vivo studies. Here, we report on a successful application of an ^225^Ac/^221^Fr generator concept and the in vivo distribution of ^221^Fr and bismuth-213 (^213^Bi).

**Methods:**

The immobilization of ^225^Ac and elution of ^221^Fr was performed on a LN2 resin column. The biodistribution of ^221^Fr and ^213^Bi was investigated in male SCID mice with LNCaP tumors at 5 and 15 min p.i.

**Results:**

Our results indicate that LN2 resin is a highly efficient resin for selective separation of ^225^Ac and ^221^Fr. The use of 0.1 M NaOAc enabled continuous elution at a constant pH. The biodistribution study revealed a fast distribution of ^221^Fr and ^213^Bi already 5 min p.i. We observed a strong accumulation of ^221^Fr to the kidneys, salivary glands and small intestine. In ^213^Bi-injected mice, the highest accumulation was in kidney and liver.

**Conclusion:**

We present an unprecedented concept utilizing LN2 resin in ^225^Ac/^221^Fr generator applications. Successfully eluted and injected ^221^Fr fractions showed strong accumulation of ^221^Fr and ^213^Bi in key organs. Our data provide preliminary evidence of the potential contribution of recoiled progeny radionuclides to side-effects in non-targeted organs.

**Supplementary information:**

The online version contains supplementary material available at 10.1007/s00259-025-07427-4.

## Introduction

Since the first U.S. Food and Drug Administration (FDA) approval of radium-223 dichloride [^223^Ra]RaCl_2_, Xofigo^®^, Bayer, Leverkusen, Germany) in 2013 [1], targeted alpha therapies (TαT) have attracted a continuous interest in the field of modern nuclear medicine. Actinium-225 (^225^Ac) has recently shown exciting clinical data when used in combination with prostate-specific membrane antigen (PSMA)-targeting radioligands such as [^225^Ac]Ac-PSMA-617 (Action, NCT04597411); [^225^Ac]Ac-PSMA I&T (TATCIST, NCT05219500); and [^225^Ac]Ac-PSMA-R2 (SatisfAction, NCT05983198). In contrast to β^−^-emitters, α-emitters release high energy (several MeV) over short tissue range (< 0.1 mm) resulting in high linear energy transfer (LET), leading to a much higher killing efficiency of cancerous cells without damaging wider surrounding healthy tissue [2]. On the other hand, unfavorable half-lives, complicated decay pathways, production, and availability issues render only a limited number of α-emitters realistically suitable for TαT [3]. Among these, thorium-227 (^227^Th, T_1/2_ = 18.7 d, E_α_ = 6.0 MeV, E_γ_ = 236 keV, I = 12.9%), ^225^Ac (T_1/2_ = 9.9 d, E_α_ = 5.8 MeV), ^224^Ra (T_1/2_ = 3.6 d, E_α_ = 5.7 MeV, E_γ_ = 241 keV, I = 4.1%), ^223^Ra (T_1/2_ = 11.4 d, E_α_ = 5.7 MeV, E_γ_ = 269 keV, I = 13.3%), bismuth-213 (^213^Bi, T_1/2_ = 45.6 min, E_α_ = 5.9 MeV, E_γ_ = 440 keV, I = 25.9%), lead-212 (^212^Pb, T_1/2_ = 10.6 h, E_β,max_ = 0.57 MeV, E_γ_ = 239 keV, I = 43.6%), and astatine-211 (^211^At, T_1/2_ = 7.2 h, E_α_ = 5.9 MeV) have been thoroughly investigated [2, 4–6].

**Fig. 1:**
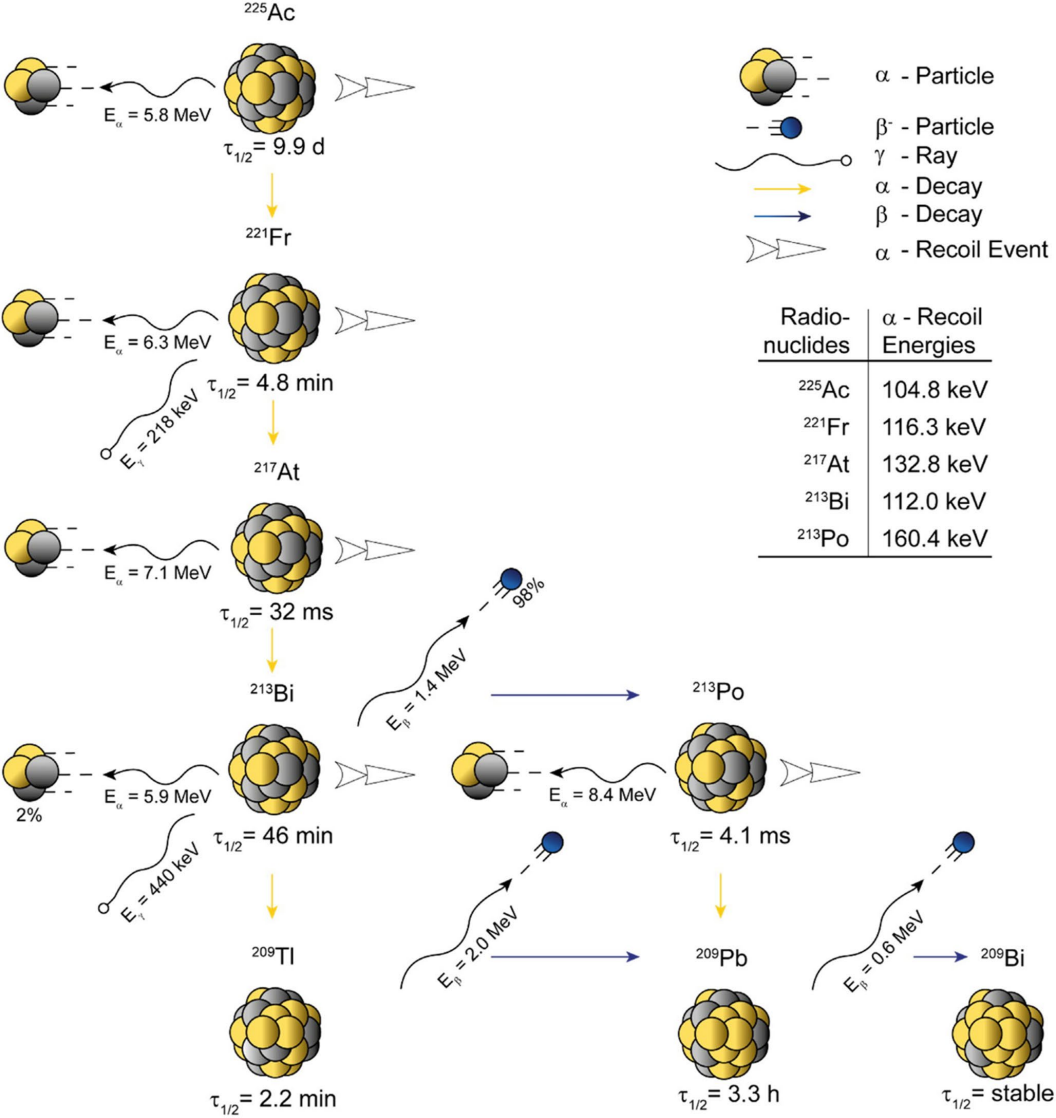
Schematic representation of the alpha in vivo nanogenerator ^225^Ac (T_1/2_ = 9.9 d, E_α_ = 5.8 MeV). The most prominent daughter radionuclide is represented by ^213^Bi (T_1/2_ = 45.6 min, E_α_ = 5.9 MeV, E_γ_ = 440 keV, I = 25.9%) which is also commonly applied for TαT. Its first daughter radionuclide ^221^Fr (T_1/2_ = 4.8 min, E_α_ = 6.3 MeV, E_γ_ = 218 keV) is used for the characterization as ^225^Ac forms so called secular equilibrium with ^221^Fr. The figure was taken from Roscher et al., 2020 [8]

In TαT, ^225^Ac, a so-called alpha in vivo nanogenerator, is currently considered to be the most promising α-emitter for pre-clinical and clinical applications. In its decay chain, a total of 28 MeV is released [7] through four dominant α-disintegrations (five in total) and two dominant β^–^-disintegrations (three in total) until the stable bismuth-209 (^209^Bi) is reached (Fig. 1). To make best use of ^225^Ac as a therapeutic agent, a thorough understanding of the in vivo distribution of ^225^Ac and its daughter radionuclides is essential for accurate dosimetry calculations. The ^225^Ac decay provides two useful γ-emissions for detection, 218 keV (I = 11.4%, francium-221 (^221^Fr), 99.9% α-branching and 0.01% β^−^-emission followed by α-decay) and 440 keV (I = 25.9%, ^213^Bi, 2.1% α-branching and 97.9% β^−^-emission followed by α-decay).

In TαT, different chelation strategies are utilized to safely transport the radioactivity to designated tumor sites within a dedicated targeting ligand. Currently, 1,4,7,10-tetraazacyclododecane-1,4,7,10-tetraacetic acid (DOTA)- and *N*,*N*’-bis[(6-carboxy-2-pyridil)methyl]−4,13-diaza-18-crown-6 (macropa)-based constructs are recognized as gold standards [9]. However, possible in vivo decomplexation or nuclear recoil phenomena can impede the intended outcomes of applied radiopharmaceuticals [10]. In case of ^225^Ac, the first daughter radionuclide ^221^Fr is not retained by the chelating agent and can diffuse away from the targeting moiety [11], especially prior to internalization. This phenomenon is even more pronounced for the downstream daughter radionuclides of ^221^Fr, particularly ^213^Bi, due to its longer half-life (45.6 min).

A biodistribution study using radionuclidically pure ^221^Fr would enable quantification of its diffusion and redistribution. For this purpose, extraction chromatography has been suggested as a suitable purification method, in which highly selective extractants fixed on an inert support, e.g. a polymer matrix or inorganic materials, serve as stationary phase in applied columns. The mobile phase usually consists of an acidic solution, e.g. HNO_3_ or HCl [12]. Previously, a new and fast generator concept has been presented to provide a ^221^Fr source from ^225^Ac [13]. Diglycolamides (TODGA, isoTODGA) were immobilized on a polyacrylonitrile (PAN) matrix and subsequently evaluated in batch and column experiments. Column experiments revealed isoTODGA-PAN and 4 M HNO_3_ to be an ideal combination of extractant and eluent for ^225^Ac sorption. Under these conditions, ^221^Fr yields of > 65% were achieved in approximately 2.5 mL of eluate with ^225^Ac contaminations below 1%, but not with the desired pH nor with sufficient amounts and purities of ^221^Fr for biodistribution studies.

In this work, we aimed to develop an ^225^Ac/^221^Fr radionuclide generator set-up for direct in vivo applications to study the biodistribution of the two main ^225^Ac progenies. Understanding the behavior of non-chelated α-emitters is crucial, as they might redistribute within the body, potentially increasing radiation risks to healthy organs and tissues. Our study seeks to provide valuable insights into their effects, ultimately contributing to a more comprehensive assessment of dosimetry and radiation safety of TαT.

## Materials and methods

### Materials

LN2 resin (G71m resin loaded with 2-ethylhexylphosphonic acid mono-2-ethylhexyl ester (HEH[EHP])) was purchased from TrisKem International having the following physical and chemical properties: particle size = 500–100 µm, density = 0.37 g/mL, capacity = 0.16 mmol/mL resin, conversion factor D_W_/k’ = 1.82).

^225^Ac was provided by Global Morpho Pharma (GMP, La Chapelle-sur-Erdre, France) as ^225^Ac(NO_3_)_3_ dry film. The full activity batch was subsequently diluted in 150 µL ultra-pure and metal-free 0.05 M HCl and transferred into a 1.5 mL Eppendorf tube (Protein LoBind^®^).

Phosphate-buffered saline (PBS, 1X, pH 7.4) was purchased from Gibco, 0.15 M sodium chloride (NaCl, pH 5.5) and sodium acetate (NaOAc) from Sigma-Aldrich. TraceMetal Grade nitric acid (HNO_3_, 67–69%) was purchased from Fischer Scientific, HIPERPUR-plus hydrochloric acid (HCl, 35%) from PanReac AppliChem, and TraceSELECT™ H_2_O from Honeywell.

### Radionuclide separation

^225^Ac(NO_3_)_3_ (~ 50 MBq, 150 µL 0.05 M HCl) was diluted with 450 µL 0.01 M HNO_3_ to a total volume of 600 µL and loaded onto 0.3 mL (Height: 19.8 mm x Diameter: 11.7 mm) LN2 column, which was pre-conditioned with at least three column volumes of 0.01 M HNO_3_. Afterwards, the column was washed with 800 µL of 0.01 M HNO_3_ (pH 2.0), 0.15 M NaCl (pH 5.5), PBS (pH 7.4), and 0.1 M NaOAc (pH 6.5). Subsequently, multiple ^221^Fr elutions were performed with 800 µL 0.1 M NaOAc (pH 6.5) after sufficient ^221^Fr in-growth time of 25–30 min. Four fractions of 150, 300, 300 and 150 µL were collected and measured immediately in a dose calibrator (ISOMED 2010, Nuvia Instruments, Dresden, Germany). Quality control was conducted retrospectively by time and energy-dependent measurements in a gamma counter (Wizard 2410, with 10 independent NaI(Tl) detectors, Perkin Elmer, Waltham, Massachusetts, USA) and a gamma spectrometer HPGe(Li) detector, Mirion Technologies, Atlanta, Georgia, USA). The activity of ^221^Fr and ^213^Bi was assessed from the photo-peaks of 218 and 440 keV, respectively.

### Cell line

LNCaP cells, derived from a human lymph node metastatic lesion of prostatic adenocarcinoma, were obtained from the German Collection of Microorganisms and Cell Cultures (Leibniz Institute DSMZ, Braunschweig, Germany).

### Animal model

For the biodistribution study, male SCID (CB-17/Icr-*Prkdc*^*scid/scid*^/Rj) mice (20–25 g, 7-weeks-old, Janvier Labs, Le Genest-Saint-Isle, France) were implanted subcutaneously (s.c.) with testosterone pellets (12.5 mg, 4 mm, prepared in-house). One day later, mice were inoculated s.c. with 5 × 10^6^ LNCaP cells into the right flank. All animal experiments were conducted in accordance with the German Animal Welfare Law and approved by the local authorities.

### Biodistribution of ^221^Fr in vivo

On Day 35 after inoculation, the mice (*n* = 3/group) were injected with a single dose of ^221^Fr and sacrificed at 5 min post-injection (p.i.). On Day 37 after inoculation, the mice (*n* = 3/group) were injected with a single dose of ^221^Fr and sacrificed at 15 min p.i. The procedure for each mouse was as follows: 25 min after the previous ^225^Ac/^221^Fr generator elution, a fresh elution of four fractions (150, 300, 300 and 150 µL) was taken and the second fraction was checked for pH (> 6.0 and < 7.0) and a 100 µL intravenous (i.v.) tail vein injection into one mouse followed within 2–3 min after elution. Standards of the injected volume were taken for each injected elution and measured in parallel. Throughout the study, a timer was continuously used to record exact time-points for all actions.

After 5–15 min the mouse was sacrificed and the following organs were collected: kidney, tumor, blood, liver, small intestine, salivary gland, large intestine, pancreas, spleen, muscle. The organs were halved or divided for pair organs to allow for two parallel samples for measurement 3–5 min after start of sectioning. One set was measured once by gamma spectrometry measurements HPGe(Li) detector. The other set of samples was measured several times in a gamma counter by NaI(Tl) crystal. The next mouse was injected ~ 50 min later.

### Biodistribution of ^213^Bi in vivo

Biodistribution of ^213^Bi was performed analogically as for ^221^Fr. On Day 35 after inoculation, the mice (*n* = 3/group) were injected with a single dose of ^213^Bi and sacrificed at 5 min p.i. On Day 37 after inoculation, the mice (*n* = 3/group) were injected with a single dose of ^213^Bi and sacrificed at 15 min p.i. For the ^213^Bi biodistributions the elutions were approximately 2 h old to not contain any ^221^Fr or ^217^At. The three mice for each time-point were injected in 5 min intervals on one day. Standards of the injected volume were taken and measured in parallel. The collection of organs and measurements was analogous to the ^221^Fr biodistribution, as described in the previous paragraph.

### Measurement HPGe(Li) detector

The gamma spectrometry measurements were performed with a HPGe(Li) detector purchased from Mirion Technologies, Atlanta, Georgia, USA. Measuring time was 5–6 min per sample. The activity of ^221^Fr and ^213^Bi were assessed from the gamma lines specifically attributed to these radionuclides.

### Measurement NaI(Tl) detector

Wizard 2410 employing 10 independent NaI(Tl) detectors was purchased from Perkin Elmer, Waltham, Massachusetts, USA. Each sample was measured six times over a period of 40–45 min, with each measurement lasting 1 min. The acquisitions were performed in the channels of 180–240 keV for ^221^Fr and 380–520 keV for ^213^Bi.

### Quantification of ^213^Bi and ^221^Fr activities in ^213^Bi- and ^221^Fr-injected mice

To determine the distribution of ^213^Bi and ^221^Fr in organs, we analyzed data from two experimental setups: mice injected with ²¹³Bi, and mice injected with ^221^Fr. We began by processing the data from the ^213^Bi-injected mice, where no ^221^Fr was present in the tissues. Thus, ^213^Bi activity was calculated by fitting an exponential decay function [Eq. (1)] to the CPM values measured in the ^213^Bi energy window, with constraints ensuring non-negative initial CPM and background.

For the ^221^Fr-injected mice, we accounted for the fact that ^213^Bi decay also contributes to the signal in the ^221^Fr window. Additionally, the calculation of ^213^Bi activity in these animals required fitting a more complex function derived from the Bateman equations.

Calibration was performed using ^225^Ac in secular equilibrium, which provided known activities for both ^213^Bi and ^221^Fr. Linear fits through the origin yielded calibration factors of *f*_*Bi*_ =1.649 +/- 0.062 Bq⁻¹ and *f*_*Fr*_ = 3.513 +/- 0.012 Bq⁻¹.

All fits were performed in OriginPro 2021 using the Levenberg–Marquardt algorithm, with instrumental weighting to account for CPM uncertainty. The calculation procedure is described in more detail in the Supplementary Material.

## Results

**Fig. 2:**
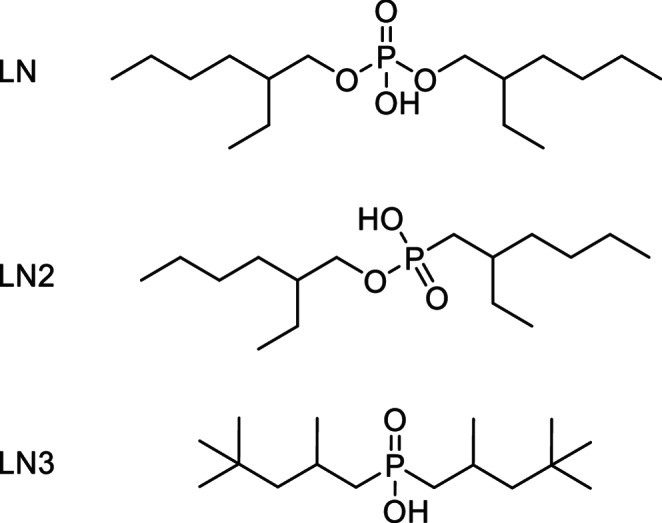
Structural details of extractants impregnated onto an inert support in the LN resin series (LN, LN2 and LN3 resins). LN resin contains bis(2-ethylhexyl) hydrogen phosphate (HDEHP), LN2 resin contains 2-ethylhexylphosphonic acid mono-2-ethylhexyl ester (HEH[EHP]) and LN3 contains bis(2,4,4-trimethylpentyl)phosphinic acid (H[TMPeP])– Cyanex272)

Focusing our literature research on resins potentially showing good retention of only Ac-225 and none of its daughter radionuclides, we focused on the three structurally different variations (LN, LN2, LN3) of the LN resin series, offering different acidities (Fig. 2) in the following decreasing order: LN > LN2 > LN3 [14]. Out of these three resins, the LN2 resin exhibited the most optimal performance in terms of ^225^Ac retention under loading and elution conditions, as well as in terms of pH, elution volume and purity of ^221^Fr.

### Elution of ^221^Fr from ^225^Ac/^221^Fr radionuclide generator

**Fig. 3:**
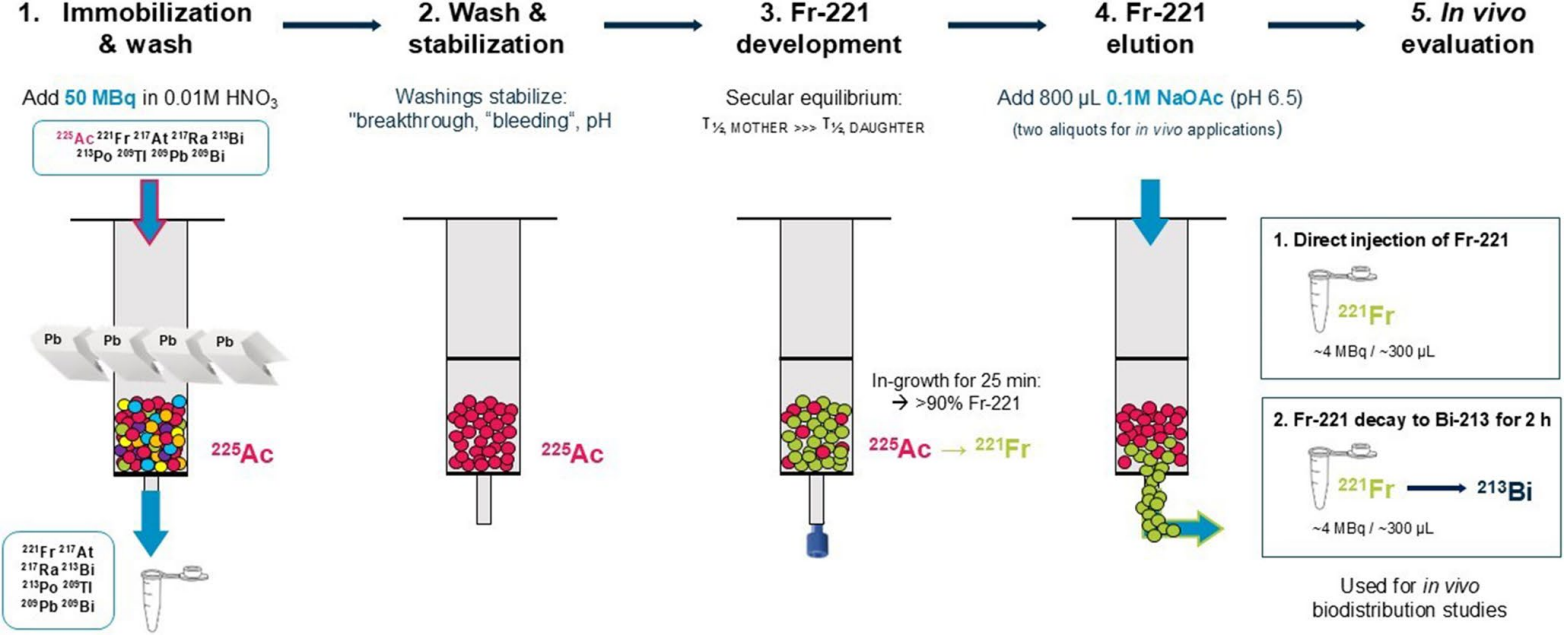
Schematic representation of ^225^Ac/^221^Fr radionuclide generator set-up. ^225^Ac(NO_3_)_3_ was loaded on a LN2 resin column and subsequently washed with 0.01 M HNO_3_ and stabilized with 0.15 M NaCl, PBS, and 0.1 M NaOAc. Upon secular equilibrium, ^221^Fr was eluted with 0.1 M NaOAc (pH 6.5) and applied for in vivo biodistribution studies without any re-formulation

^225^Ac(NO_3_)_3_ in 0.01 M HNO_3_ (~ 50 MBq, 600 µL, pH 1.0) was manually loaded on a LN2 resin column (V_c_ = 300 µL) and subsequently washed with 0.01 M HNO_3_ (900 µL) to clear the column of all ^225^Ac progeny radionuclides (^221^Fr, ^217^At, ^217^Ra, ^213^Bi, ^213^Po, ^209^Tl, ^209^Pb, ^209^Bi). In order to obtain ^221^Fr fractions with a more physiological pH which can be tolerated by mice upon injection, eluents with a pH range of 6.5 to 8.5 were chosen. Therefore, the column was manually washed with an elution volume (V_E_) of 800 µL of each 0.15 M NaCl (pH 5.5), PBS (pH 7.4) and 0.1 M NaOAc (pH 6.5) manually after loading with ^225^Ac (Fig. 3).

In our experiments, 0.1 M NaOAc proved to be an ideal eluent, maintaining the column at a physiologically relevant pH of approximately 6.5, which was essential for downstream in vivo applications. This buffer system not only ensures chemical stability but also significantly minimizes early ^225^Ac “breakthrough” and “bleeding” phenomena, which are critical for achieving high radionuclidic purity of the eluted ^221^Fr. After an in-growth time of 25 min, manual elution of ^221^Fr fractions with 0.1 M NaOAc (V_E_ = 800 µL) was consistently feasible up to eight times per day, enabling a practical and reproducible workflow. Furthermore, we have assessed the purity of 24-hour-old eluates and found that in all but one instance (out of 18 eluates analyzed), both ^221^Fr and ^213^Bi were below the lower limit of quantification (LLOQ), indicating only minimal ^225^Ac breakthrough. However, a notable disadvantage of using NaOAc was a reduced elution efficiency, with a decrease in ^221^Fr stripping yield of approximately 20% compared to stronger mineral acids as eluents.

Still, high ^221^Fr elution yields of > 50% in comparison to immobilized ^225^Ac were possible with ^225^Ac contaminations remaining significantly low (< 0.1%). The eluted ^221^Fr activity would have been sufficient for injecting three mice per time-point while obtaining enough signal for the measurement upon all planned biodistribution studies. Based on the short half-life of ^221^Fr and resulting handling challenges, we decided to inject a single mouse per elution.

### Biodistribution of ^221^Fr in ^221^Fr-injected mice

**Fig. 4:**
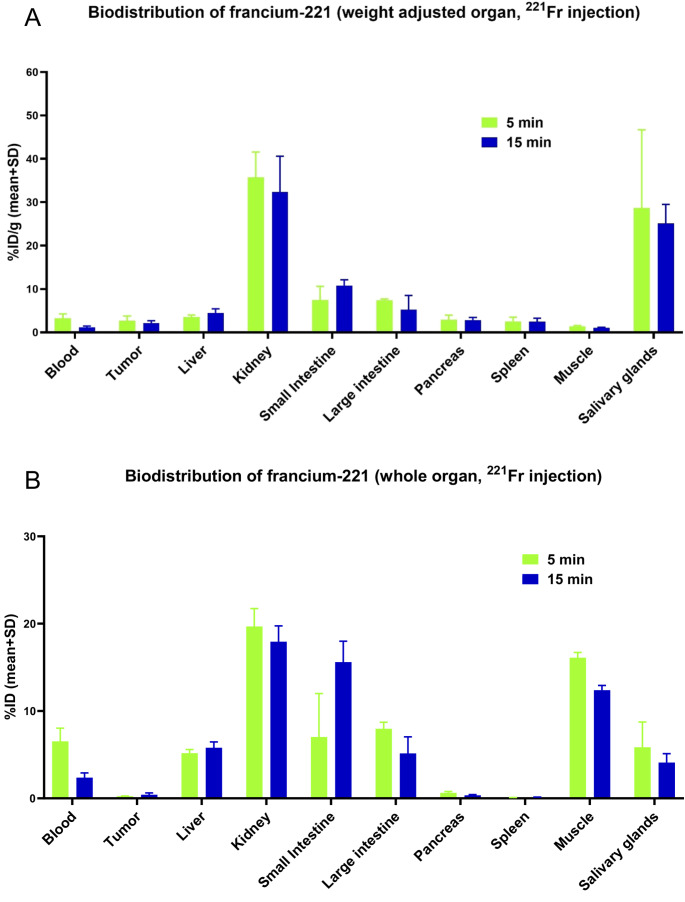
Biodistribution data from injection of ^221^Fr in LNCaP tumor bearing mice. Results obtained from measuring with a NaI(Tl) detector depicted in (**A**) percentage of injected dose per gram organ or tissue (**B**) and as percentage of injected dose per organ or tissue. The values are mean values with standard deviation (*n* = 3 per time-point) considering linear propagation of uncertainties

Due to its short half-life, measuring the biodistribution of ^221^Fr presents significant challenges. Organ samples were divided in half and simultaneously measured with both the HPGe detector (Supplementary Material, Figure S1) and the NaI(Tl) detector (Fig. 4). This strategy enabled the acquisition of complementary data, combining the HPGe detector’s superior energy resolution for precise radionuclide identification with the NaI(Tl) detector’s higher detection efficiency.

The data from the NaI(Tl) detector showed a very rapid distribution of free ^221^Fr after injection and at 5 min p.i., only 3.2% of the injected dose per g tissue (ID/g) of ^221^Fr was still detected in the blood, which decreased further to 1.2% ID/g at the 15-min time-point (Fig. 4A). The kidneys showed the highest activity accumulation, with 35.7% and 32.4% ID/g at 5 and 15 min p.i., respectively. The salivary glands exhibited 28.7% and 25.1% ID/g (5 and 15 min p.i.), while the small intestine showed an accumulation of 7.5% and 10.8% ID/g (5 and 15 min p.i.). Also, the large intestine showed an accumulation of 7.4% and 5.2% ID/g (5 and 15 min p.i.). Other organs and tissues displayed minimal to no accumulation.

Analyzing the biodistribution data considering the entire organ, a more complete picture of where the ^221^Fr accumulated in a mouse can be observed (Fig. 4B). Specifically, 19.7% ID accumulated in the kidney after 5 min and retained 18.0% ID after 15 min. The small intestine initially retained 7.0% ID after 5 min, which increased to 15.6% ID after 15 min. The whole muscle calculated weight exhibited relatively high ^221^Fr accumulation with 16.1% ID and 12.4% ID (5 and 15 min p.i.). The uptake values observed in muscle may primarily reflect the tissue’s vascularization rather than true cellular uptake by myocytes, especially considering the rapid blood clearance of ^221^Fr and ^213^Bi and their potential redistribution during circulation. Nevertheless, these findings are important, as they suggest a possible source of off-target toxicity that has not been previously considered in ^225^Ac-based TαT. Some bias is also given with higher accumulation in organs with higher mass, however, even a smaller organ, such as the salivary glands, accumulated 5.9% ID within 5 min of the whole injected activity of ^221^Fr and retained 4.1% ID after 15 min. This trend was also confirmed by measurements using the HPGe detector (Supplementary Material, Figure S1).

### Biodistribution of ^213^Bi in vivo in ^213^Bi-injected mice

**Fig. 5:**
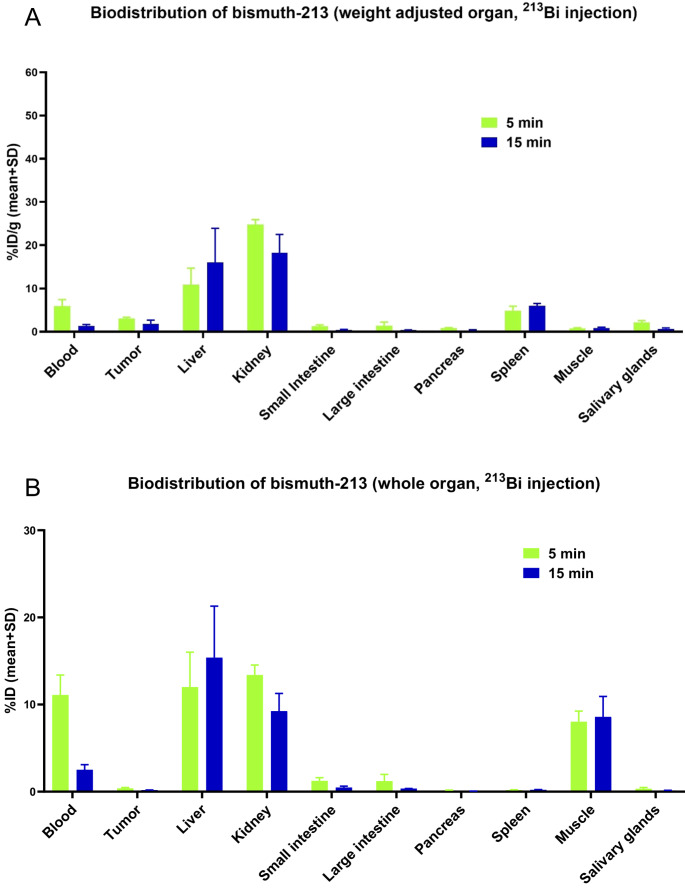
Biodistribution data from injection of ^213^Bi in LNCaP tumor bearing mice. Results obtained from measuring with a NaI(Tl) detector depicted in (**A**) percentage of injected dose per gram organ or tissue (**B**) and as percentage of injected dose per organ or tissue. The values are mean values with standard deviation (*n* = 3 per time-point) considering linear propagation of uncertainties

Similarly, for the biodistribution of ^213^Bi, the organ samples were divided in half and measured simultaneously using the HPGe detector (Supplementary Material, Figure S2) and the NaI(Tl) detector (Fig. 5). The distribution of ^213^Bi in the mice was fast, with only 5.9% ID/g remaining in the blood at 5 min and 1.4% ID/g at 15 min (Fig. 5A). The kidneys exhibited 24.8% ID/g at 5 min and 18.3% ID/g at 15 min. The liver showed an accumulation of 10.9% ID/g at 5 min, which increased to 16.0% ID/g at 15 min. The spleen showed some accumulation, with 4.9% ID/g at 5 min and 6.0% ID/g at 15 min. No significant accumulation of ^213^Bi was observed in the other organs and tissues.

When analyzing the biodistribution data across the entire organ, higher accumulation was observed primarily in the blood, liver, and muscle (Fig. 5B). Specifically, 12.0% ID accumulated in the liver at 5 min and retained 15.4% ID after 15 min. An initial 13.4% ID detected in the kidneys at 5 min and decreased to 9.2% ID after 15 min. In the blood, 11.1% ID was present 5 min after injection, but declined to 2.5% ID after 15 min.

### Biodistribution of ^213^Bi in vivo in ^221^Fr-injected mice

**Fig. 6:**
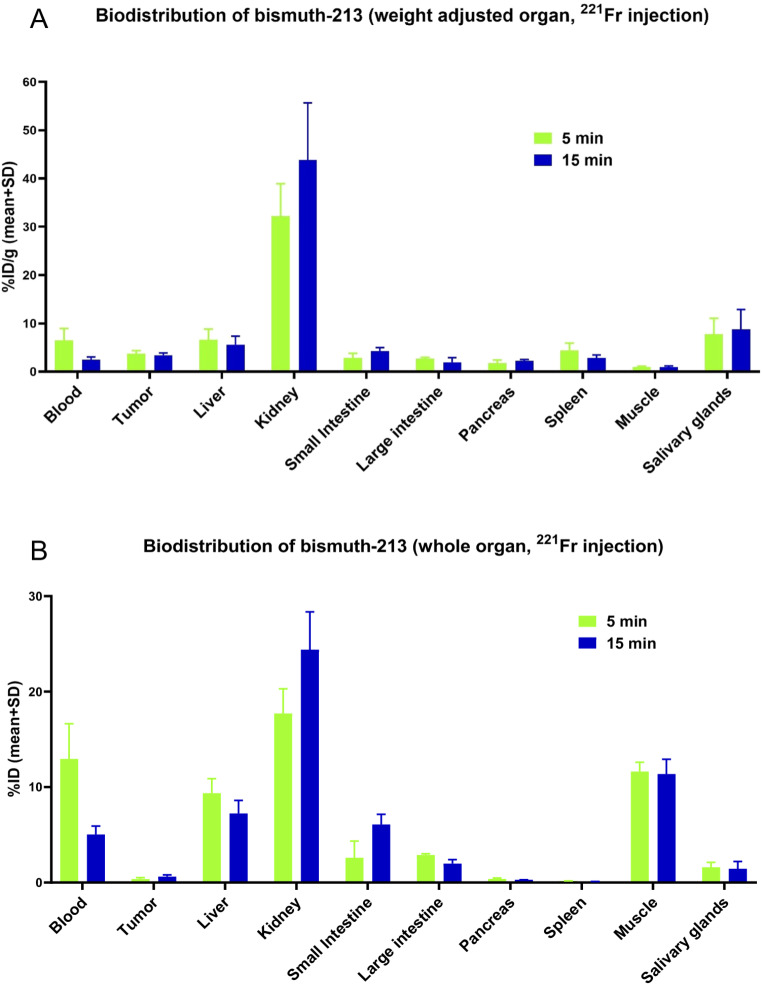
Biodistribution of ^213^Bi from injection of ^221^Fr in LNCaP tumor bearing mice. Results obtained from measuring with a NaI(Tl) detector depicted in (**A**) percentage of injected dose per gram organ or tissue (**B**) and as percentage of injected dose per organ or tissue. The values are mean values with standard deviation (*n* = 3 per time-point) considering linear propagation of uncertainties

The following study focused on the accumulation of ^213^Bi generated from ^221^Fr injections into the mice. Subsequently, these results were compared with data of direct ^213^Bi injections (Fig. 6). Only 6.5% ID/g of ^213^Bi was detected in the blood at 5 min, which decreased to 2.5% ID/g at 15 min (Fig. 6A). The kidneys showed accumulation of 32% ID/g at 5 min, increasing to 44% ID/g at 15 min. The salivary glands exhibited some accumulation, with 7.7% ID/g at 5 min and 8.8% ID/g at 15 min. The liver showed 6.6% ID/g at 5 min and 5.6% ID/g at 15 min. The spleen demonstrated 4.4% ID/g at 5 min, which decreased to 2.9% ID/g at 15 min. The small intestine showed accumulation of 2.9% ID/g at 5 min, increasing to 4.2% ID/g at 15 min. No significant accumulation of ^213^Bi was observed in the other organs and tissues.

**Fig. 7:**
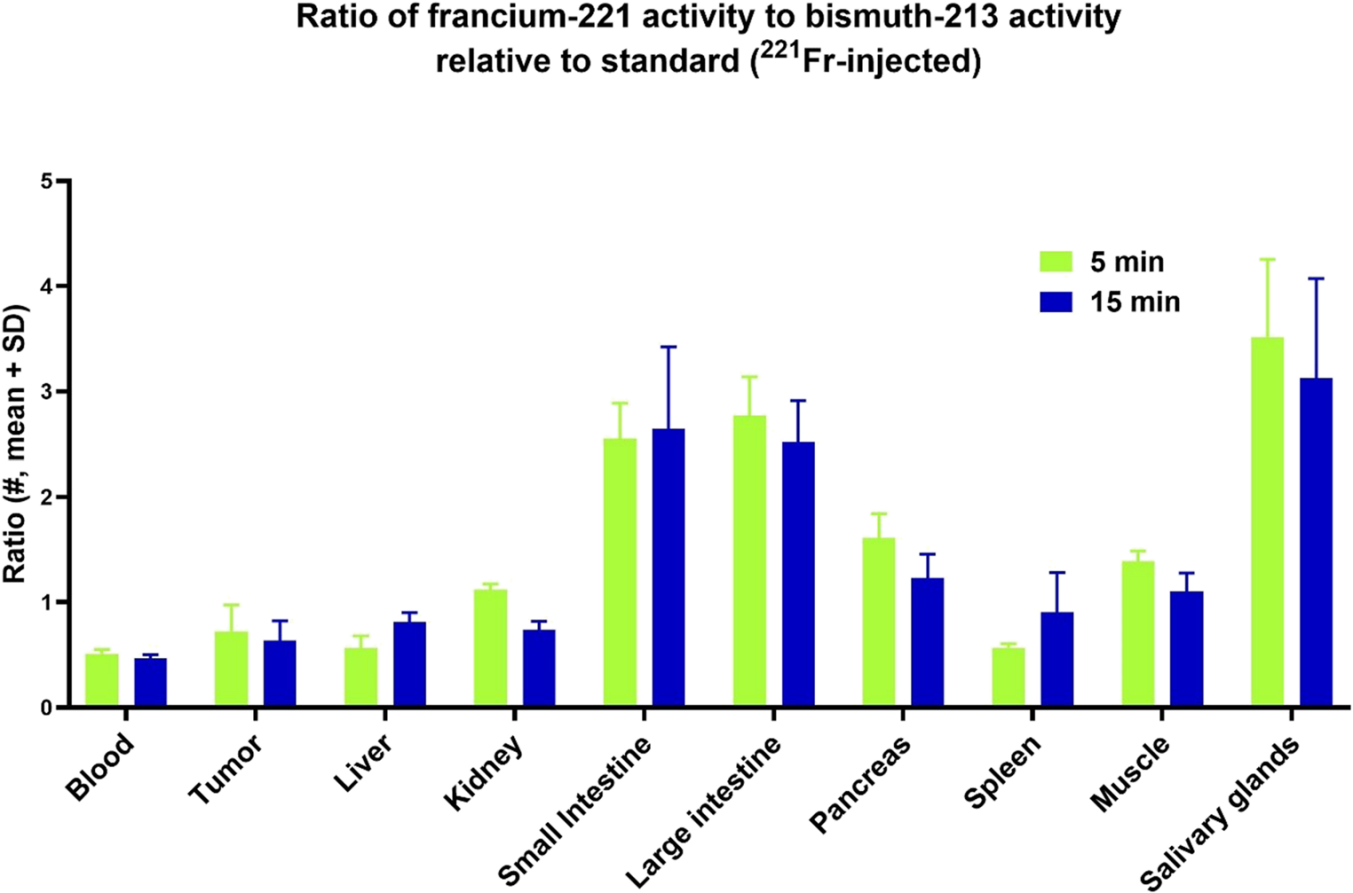
^221^Fr/^213^Bi activity ratio in the organs of LNCaP tumor-bearing mice, measured with a NaI(Tl) detector, compared to a reference sample undergoing physical decay only

To elucidate the organ-specific retention of ^221^Fr and ^213^Bi, we compared their activity ratios in various mouse organs to the identical ratio of a standard sample undergoing physical decay only (Fig. 7). This comparison assumes that if ^221^Fr and ^213^Bi had the same biodistribution across all organs, the plotted activity ratios would equal 1.00 for all organs (^221^Fr = ^213^Bi). Ratios greater than 1.00 would indicate organs of a higher affinity for ^221^Fr (^221^Fr > ^213^Bi), while ratios lower than 1.00 would suggest a preference for ^213^Bi (^221^Fr < ^213^Bi).

The highest ratios were observed in the small intestine (2.55 and 2.64 at 5 and 15 min), the large intestine (2.77 and 2.52 at 5 and 15 min), and the salivary glands (3.51 and 3.13 at 5 and 15 min), respectively. In these organs, the elevated ratios indicate a strong preferential retention of ^221^Fr over ^213^Bi. In contrast, a higher affinity for ^213^Bi is observed in the blood and liver. However, the ratios observed in the blood are not indicative of ^221^Fr or ^213^Bi affinities. The retention in all organs significantly decreased the activity concentration in the blood. This suggests that rapid clearance from circulation, rather than strong binding to blood components [15], is the predominant factor influencing blood activity levels. For the remaining organs, no significant difference in affinity between ^221^Fr and ^213^Bi was observed.

## Discussion

Our establishment of a new ^225^Ac/^221^Fr radionuclide generator allows the elution of ^221^Fr (and the follow up daughter ^213^Bi) under physiological conditions suitable for injection into a living organism and represents a crucial step towards understanding the behavior and biodistribution of non-complexed radionuclides in mammals. The observation that daughter radionuclides produced by alpha emitters do not remain at the site of the mother radionuclide led to the hypothesis that daughter radionuclides contribute not only to the therapeutic efficacy but also appear to play a role in adverse events. A publication by Kratochwil et al. described high-resolution quantitative Positron Emission Tomography (PET) imaging with [^68^Ga]Ga-PSMA-617 while extrapolating radiation dosimetry for [^213^Bi]Bi-PSMA-617 and comparing its therapeutic index with [^225^Ac]Ac-PSMA-617 [16]. Under the assumption of homogenous dose distribution, equivalent doses for critical organs and tumor lesions exhibited notable differences in dose distributions to off-target organs. In contrast to [^213^Bi]Bi-PSMA-617 (1.2 GBq), [^225^Ac]Ac-PSMA-617 (7.4 MBq) delivered higher tumor doses (42.1 vs. 7.6 Sv_RBE5_), also resulting in significantly higher salivary gland exposure (17.0 vs. 9.7 Sv_RBE5_). This near two-fold dose to salivary glands is hypothesized to stem from the complex decay chain of ^225^Ac.

Earlier studies investigated the biodistribution of ^221^Fr and ^213^Bi following treatment with an ^225^Ac-labeled antibody which acted as an in vivo generator for ^221^Fr and ^213^Bi. Song et al. used an anti-HER-2/neu monoclonal antibody in HER-2/neu transgenic mice and found not only very good therapeutic efficacy but also accumulation of ^221^Fr and ^213^Bi in the kidneys, which probably contributed to the long-term renal toxicity observed in the surviving mice [17]. Miederer et al. administered high activities of an ^225^Ac-labeled IgG3 antibody and showed redistribution of ^221^Fr and ^213^Bi to the kidney, small intestine and, to a lesser extent, the stomach [18]. Similarly, Schwartz et al. found that non-equilibrium ^213^Bi from a ^225^Ac-labeled huM195 antibody contributes a significant fraction of the total radiation dose to the kidney [19].

While these studies provide insights on the fate of the daughter radionuclides, they are hampered by the set-up of using an antibody as an in vivo generator for ^221^Fr and ^213^Bi. The antibody itself exhibited a specific pharmacokinetic distribution pattern, with the location of the in vivo generators/antibodies constantly changing. Additionally, the antibody also supplied a constant rate of ^221^Fr and ^213^Bi over the whole study making it hard to distinguish between an ^221^Fr or ^213^Bi generated in the organ vs. an ^221^Fr or ^213^Bi redistributed into the organ. From an experimental perspective, conducting biodistribution studies with ^221^Fr is a significant challenge due to its short half-life. As an alternate approach, the less accessible ^227^Ac (T_1/2_ = 21.7 years) could be employed instead, as its first progeny ^223^Fr has a half-life approximately four times longer than ^221^Fr used in this study [20]. This extended half-life of 22 min would potentially improve the feasibility of experimental studies on francium’s biochemical properties. Another strategy involved the cesium-132 (^132^Cs) isotope, a same-group element, with a reasonable half-life of T_1/2_ = 6.48 h [21]. In mice, the uptakes of ^132^Cs to kidneys and blood (33.0 ± 7.7; 0.48 ± 0.05% ID/g) were much lower compared to ^221^Fr-based biodistribution studies (52.3 ± 8.4; 5.4 ± 0.3% ID/g) [22] indicating that ^132^Cs is not biochemically analogous to ^221^Fr.

To overcome these limitations and enable the investigation of the distribution characteristics of ^221^Fr or ^213^Bi a new type of a radionuclide generator was needed. However, the short half-life of ^221^Fr (T_1/2_ = 4.8 min) offers distinct operational challenges of such generators, additionally calling for novel and innovative concepts for proper ^221^Fr retrieval.

Yuan et al. designed and set up one of the first ^225^Ac/^221^Fr generator concepts [23]. For this purpose, a DOTA-biotin construct was radiolabeled with ^225^Ac, subsequently reacted with an immobilized avidin column and eluted with 2 mL of sterile saline to yield desired ^221^Fr fractions. However, the utilization of such generator principles for short-lived radionuclides is not favored due to the difficult operation, complexity, and overall cost inefficiency.

Aside from amide-based resins such as diglycoamide (DGA), organophosphorous-based materials have proven efficient in the extraction and separation of actinides. Their phosphorus-oxygen (P = O) and phosphorus-sulfur (P = S) centers of high polarity and charge density allow the formation of complexes with desired f-block elements, even in complex mixtures [24]. Furthermore, the extractants can be easily tuned by side chain modifications for improved solubility, lipophilicity, and selectivity [25].

Extraction chromatographic resins based on carbamoylphosphine oxide (CMPO) dissolved in tri-*n*-butyl phosphate (TBP) [26] and di(2-ethylhexyl)orthophosphoric acid (HDEHP) [14] have proven to successfully immobilize ^225^Ac^3+^ ions, which have not been utilized in ^221^Fr separation procedures yet. In extraction chromatographic studies of ^225^Ac and various recoil effect elements (REE), Ostapenko et al. have concluded that the optimal ^225^Ac sorption conditions in LN resins are achieved in diluted nitric acid (0.05 M HNO_3_), as opposed to TRU (2–4 M HNO_3_) and DGA resins (4–7 M HNO_3_) [27]. With respect to these observations, we chose to work with the LN resin series for our ^225^Ac/^221^Fr radionuclide generator studies.

Other than the above-mentioned TRU and DGA resins, which are based on neutral extractants– thus requiring high HNO_3_ concentrations for strong retention of Ac– the LN resin series is based on liquid cation exchangers. Accordingly, the extraction of cations such as Ac depends on the pH of the aqueous solution, with higher pH values leading to stronger retention. This makes this type of resin particularly suitable in this case as the ^225^Ac needs to remain retained on the resin while ^221^Fr is eluted under near neutral conditions for direct in vivo application.

We aimed to evaluate a novel proof-of-concept for a ^225^Ac/^221^Fr generator and to investigate the biodistribution profiles of free ^221^Fr and ^213^Bi to potentially explain off-target effects in TαT. Overall, our data confirmed the accumulation of ^221^Fr and ^213^Bi in various organs. The first observation from our studies is the rapid clearance of the injected ^221^Fr and ^213^Bi from blood. Only 6.5% of the injected activity of ^221^Fr (decay corrected) remains in the blood after 5 min, whereas ^213^Bi is somewhat slower with 11.1% ID remaining in the blood. The summation of the remaining ^221^Fr activity of all organs gives a value of 69.3% and showed that 30.7% of the ID has been cleared from the body within 5 min of application. For ^213^Bi we could show that 51.7% ID are eliminated and 48.3% ID are retained in various organs. With these numbers in mind, it seems likely that a certain amount of ^221^Fr and ^213^Bi generated from ^225^Ac in the blood is eliminated rapidly from the body.

As previously suggested by studies using ^225^Ac as an in vivo generator, our data clearly show that the major organ of accumulation for ^221^Fr as well as ^213^Bi were the kidneys. Both radionuclides are cleared most likely *via* renal elimination, but seemed to be retained to a certain extent. Potentially ^213^Bi could be retained in the kidney bound to a bismuth-metal binding protein, which also binds stable ^209^Bi [28]. If this protein is also binding and retaining ^221^Fr or it is a different mechanism, this remains to be investigated.

Using our ^225^Ac/^221^Fr generator to study the distribution of ^213^Bi, we could clearly show that ^213^Bi accumulates in the liver. This is in contrast to other test systems, such as those using polymersomes as model carriers, which naturally accumulate in the liver [29]. A study of chronic ^209^Bi exposure in rats also showed accumulation of ^209^Bi in the liver of normal rats [30], suggesting a physiological mechanism responsible for ^213^Bi accumulation in the liver of mammals.

The distribution of ^221^Fr in the mouse showed, in addition to the kidney, the highest % ID/g in salivary glands, with elevated levels also in the small intestine and slightly lower levels in the large intestine. ^221^Fr exhibited 2.5 times higher accumulation in the small and large intestines and more than 3 times higher accumulation in the salivary glands in comparison to ^213^Bi. Specifically, the accumulation of ^221^Fr in the salivary glands could be of importance for targeted radionuclide therapy. The currently used ^225^Ac-labeled low-molecular-weight PSMA ligands show per se salivary gland accumulation and this toxicity could be enhanced with non-PSMA-targeted ^221^Fr accumulating in the salivary gland. If this ^221^Fr accumulation could be translated into humans, it would have implications for any long-circulating ^225^Ac-labeled ligands, as this would act as a generator for additional ^221^Fr.

In our research on both the distribution of ^221^Fr and ^213^Bi, we used the opportunity to also investigate the re-distribution of ^213^Bi, which was generated from ^221^Fr. Overall, this distribution pattern exhibited similarities that resembled those of ^221^Fr. ^221^Fr accumulated in the small intestine, large intestine, and salivary glands, whereas no direct accumulation of ^213^Bi was observed in these organs. Instead, the presence of ^213^Bi in these tissues is most likely due to the decay of accumulated ^221^Fr. This is supported by observations that ^213^Bi clears from the blood at a rate characteristic of ^221^Fr. Both radionuclides accumulated in the kidneys, so no conclusion can be drawn from the kidney values.

A major challenge for the future will be the translatability of the in vivo generated mouse data to patients. Data generated with ^225^Ac-labeled antibodies in non-human primates have shown accumulation of ^213^Bi in the kidneys [31]. Also, SPECT imaging undertaken in the course of clinical studies have been able to measure ^213^Bi and ^221^Fr in patients treated with ^225^Ac-labeled compounds. However, the signal for imaging is fairly low and from an experimental perspective, conducting studies of ^221^Fr is a significant challenge due to its short half-life.

## Conclusion

In this study, we gained valuable insights into the biodistribution patterns of ^225^Ac progenies, specifically ^221^Fr and ^213^Bi, in various organs and tissues. Our findings suggest that these progenies rapidly distribute from the blood into their preferred organ and may contribute to the increased side-effects observed in TαT. Notably, ^221^Fr exhibited a strong affinity for kidneys, salivary glands and both small and large intestine, while ^213^Bi selectively accumulated in the kidneys and liver. These observations are valid for the distribution of free ^221^Fr and ^213^Bi from the blood to organs and enhance our understanding of the potential additive side-effects of TαT and underscore the importance of accurate dosimetry in therapies involving complex alpha-emitting in vivo nanogenerators, such as ^225^Ac. Using ^225^Ac-labeled compounds with distinct pharmacokinetics will, of course, influence the distribution of free ^221^Fr and ^213^Bi depending on the organ in which they are generated. Therefore, this re-distribution has to be investigated individually for each compound.

## Electronic supplementary material

Below is the link to the electronic supplementary material.

ESM 1 (DOCX 383 KB)

## Data Availability

The datasets generated during an/or analyzed during the current study are available from the corresponding author on reasonable request.
