# Supplementary material for: Biodistribution of free Francium-221 and Bismuth-213 in Tumour-bearing SCID mice after successful development of Actinium-225/Francium-221 radionuclide generator Set-up
Source: Eur J Nucl Med Mol Imaging. 2025 Jul 1;53(1):633–46. doi: 10.1007/s00259-025-07427-4 (PMC12660422; doi:10.1007/s00259-025-07427-4)
Supplement: Supplementary file 1 — (DOCX 383 KB) [file 259_2025_7427_MOESM1_ESM.docx]

**Biodistribution of Free Francium-221 and Bismuth-213 in Tumour-bearing SCID Mice after Successful Development of Actinium-225/Francium-221 Radionuclide Generator Set-up**

Sabine Zitzmann-Kolbe^1^, Yvonne Remde^2^, Ingrid Moen^3^, Balázs Madas^4^, László Mázik^4,5^, Frans Suurs^3^, Steffen Happel^6^, Martin Schäfer^2^, Christoph Schatz^1^, Harun Taş^7^, Urs B. Hagemann^1^, Martina Benešová-Schäfer^7,*^

1. Bayer AG, Berlin, Germany

2. Service Unit for Radiopharmaceuticals and Preclinical Studies, German Cancer Research Center (DKFZ), Im Neuenheimer Feld 280, 69120 Heidelberg, Germany

3. Bayer AS, Oslo, Norway

4. Environmental Physics Department, Institute for Energy Security and Environmental Safety, HUN-REN Centre for Energy Research, Konkoly-Thege Miklós út 29-33, 1121 Budapest, Hungary

5. Doctoral School of Physics, ELTE Eötvös Loránd University, Pázmány Péter sétány 1/A, 1117 Budapest, Hungary

6. TrisKem International, 3 rue des Champs Geons, 35170 Bruz, France

7. Research Group Translational Radiotheranostics, German Cancer Research Center (DKFZ), Im Neuenheimer Feld 280, 69120 Heidelberg, Germany

**E-Mail addresses**:

sabine.zitzmann-kolbe@life-mi.com; y.remde@dkfz-heidelberg.de; ingrid.moen@bayer.com; balazs.madas@ek.hun-ren.hu; laszlo.mazik@ek.hun-ren.hu; frans.suurs@bayer.com; shappel@triskem.fr; martin.schaefer@dkfz-heidelberg.de; christoph.schatz@bayer.com; harun.tas@dkfz-heidelberg.de; urs.hagemann@bayer.com; m.benesova@dkfz-heidelberg.de

***Correspondence to**:

Dr. Martina Benešová-Schäfer

German Cancer Research Center (DKFZ)

Foundation under Public Law

Im Neuenheimer Feld 280

69120 Heidelberg

Germany

E-mail: [m.benesova@dkfz-heidelberg.de](mailto:m.benesova@dkfz-heidelberg.de)

Phone: +49-6221-42-5355

Fax: +49-6221-42-5356

**Running title**: *In vivo* Fr-221 and Bi-213 biodistribution

**Supplement data:**

**Calculation of ^213^Bi distribution in ^213^Bi-injected mice**

In order to determine the activities, we focused first on the count per minute (CPM) values in the ^213^Bi window channels of the NaI(Tl) detector for ^213^Bi-injected mice. In these experiments, there is no ^221^Fr in the organs, so the initial CPM value of ^213^Bi can be determined by fitting the following exponential decay curve to the CPM data as the function of time:

${CPM}_{Bi-213}\left( t \right)={CPM}_{Bi-213}\left( 0 \right)\times e^{-\frac{t}{\tau_{Bi-213}}}+b_{Bi-213}$, (1)

where *CPM_Bi-213_*(*t*) and *CPM_Bi-213_*(0) are the CPM values at time *t* (measured) and *t* = 0 (fitted), *τ_Bi-213_* is the mean lifetime of ^213^Bi, and *b_Bi-213_* is the background. The fitting was done with the constraints that both *CPM_Bi-213_*(0) and *b_Bi-213_* are ≥ 0. While the organs of a given mouse were measured at the same time, the standard was measured 98.6 s later in case of each mouse, which had to be considered.

A calibration was performed using ^225^Ac in secular equilibrium with its progeny with known activity. CPM values in the ^213^Bi window were plotted as the function of the equilibrium activity of ^213^Bi. Next, a linear function was fitted on the data with an intercept at 0 resulting in a slope of *f_Bi_* =1.649 +/- 0.062 Bq^-1^. Using this value, the activity of ^213^Bi in different organs was calculated at time-points of sectioning. In the case of the standard, there were no independent measurements.

OriginPro 2021 (64-bit) 9.8.0.200 (Academic) was used for fitting with the Levenberg-Marquardt iteration algorithm. The uncertainty of the CPM values was considered by instrumental weighting. The uncertainty of time was considered to be zero. These apply not only to ^213^Bi-injected mice, but also for ^211^Fr and ^213^Bi data of ^221^Fr-injected mice (see the next two sections).

Activity concentrations were calculated by dividing the measured activity by the organ sample weight. Total organ activities were calculated by multiplying activity concentrations by the whole organ weight, assuming homogeneous activity distribution in the organs.

Due to measurement limitations, the total weight of the blood and muscles was estimated to be 7.8% (blood) and 45.0% (muscles) of total body weight, in line with established/reported parameters.

Organ weight uncertainties (for both samples and whole organs) were considered to be negligible. This allowed for the relative uncertainties of the activity concentrations and total activities to equal to the corresponding relative uncertainties of the measured activities. The uncertainty calculations were conducted assuming a linear propagation of uncertainties.

The percentage of injected dose (%ID) was calculated as the time-adjusted total activity in an organ relative to the injected activity. The percentage of injected dose per gram (%ID/g) was derived by dividing this value by the total organ weight.

These apply not only to ^213^Bi-injected mice, but also for ^211^Fr and ^213^Bi data of ^221^Fr-injected mice (see the next two sections).

**Calculation of ^221^Fr distribution in ^221^Fr-injected mice**

The ^221^Fr activities for ^221^Fr-injected mice were determined through CPM values in the ^221^Fr window channels of the NaI(Tl) detector. In these experiments, both ^221^Fr and ^213^Bi were detected in the organs. Furthermore, analogous experiments with ^213^Bi revealed a substantial overlap of ^221^Fr window channels

To correct the overlapping signals, corrections were mandatory to obtain an unbiased estimate of ^221^Fr decays. For this purpose, the CPM data of the three standards in the ^221^Fr experiments were used. The CPM ratio in the ^221^Fr and ^213^Bi windows was calculated by fitting a linear function considering the uncertainties of CPM values in both windows. We concluded that per single CPM in the ^213^Bi window an additional 0.2753 +/- 0.0074 CPM in the ^221^Fr window had to be taken into consideration:

${CPM}_{Fr-221, corrected}(t)={CPM}_{Fr-221, measured}(t)-0.2753\times{CPM}_{Bi-213}(t)$. (2)

After this correction, the initial, corrected CPM values of ^221^Fr were determined by fitting an exponential decay curve to the time-dependent data. In the following, *CPM_Fr-221_* refers to *CPM_Fr-221, corrected_* as defined by equation (2):

${CPM}_{Fr-221}\left( t \right)={CPM}_{Fr-221}\left( 0 \right)\times e^{-\frac{t}{\tau_{Fr-221}}}+b_{Fr-221}$, (3)

where *CPM_Fr-221_*(*t*) and *CPM_Fr-221_*(0) are the CPM values at time *t* (measured and corrected) and *t* = 0 (fitted), *τ_Fr-221_* is the mean lifetime of ^221^Fr, and *b_Fr-221_* is the background. The fitting was done with the constraints that both *CPM_Fr-221_*(0) and *b_Fr-221_* are ≥ 0. While the organs of a given mouse were measured at the same time, the standard was measured 98.6 s later in case of each mouse, which had to be considered.

A calibration was performed using ^225^Ac in secular equilibrium with its progeny of known activity. In analogy, a correction of CPM values was necessary due to the overlap of ^213^Bi decay into ^221^Fr window channels.

The corrected CPM values were plotted as the function of the equilibrium activity of ^221^Fr. Next, a linear function was fitted on the data with an intercept at 0, resulting in a slope of *f_Fr_* = 3.513 +/- 0.012 Bq^-1^the activity of ^221^Fr in different organs was calculated at time-points of sectioning. The mean values of three independent measurements were calculated. Uncertainties were calculated based on assumption of linear propagation.

**Calculation of ^213^Bi distribution in ^221^Fr-injected mice**

The distribution of ^213^Bi in ^221^Fr-injected mice was determined using the Bateman equation. The half-life of ^217^At is several orders of magnitude shorter than the half-lives of ^221^Fr and ^213^Bi. Therefore, a simplified equation can be used for fitting CPM values in the ^213^Bi window in case of ^221^Fr-injected mice:

${CPM}_{Bi-213}\left( t \right) ={CPM}_{Bi-213}\left( 0 \right)\times e^{-\frac{t}{\tau_{Bi-213}}}+{CPM}_{Fr-221}\left( 0 \right)\times\frac{\tau_{Fr-221}}{\tau_{Fr-221}-\tau_{Bi-213}}\times\frac{f_{Bi}}{f_{Fr}}\times\left( e^{-\frac{t}{\tau_{Fr-221}}}-e^{-\frac{t}{\tau_{Bi-213}}}\times\frac{\tau_{Bi-213}\times\left( 1-e^{-\frac{\Delta t}{\tau_{Bi-213}}} \right)}{\tau_{Fr-221}\times\left( {1-e}^{-\frac{\Delta t}{\tau_{Fr-221}}} \right)} \right)+b_{Bi-213}$ (4)

where *CPM_Bi-213_*(*t*) and *CPM _Bi-213_*(0) are the *CPM* values at time *t* (measured) and *t* = 0 (fitted), *CPM_Fr-221_*(0) is a fixed parameter taken from equation (1), τ*_Bi-213_* and τ*_Fr-221_* are the mean lifetimes of ^213^Bi and ^221^Fr, Δ*t* is 60 s, *f_Bi_* and *f_Fr_* are the calibration factors, and *b_Bi-213_* is the background which is the second fitted parameter. The fitting was done with the constraints that both *CPM_Bi-213_*(0) and *b_Bi-213_* are ≥ 0.

**Biodistribution of ^221^Fr *in vivo***

Tissue masses used to convert the injected dose per gram of tissue to the injected dose per whole tissue were estimated based on the total body weight of each mouse and organ-specific percentage values. The following fixed percentages of total body weight were applied: 6% for total bone mass, 45% for skeletal muscle, 7.8% for total blood volume, and 18% for skin. All other organs were considered individually, using their measured weights for calculations.

**Example of Ge(Li) spectra with the corresponding peaks**


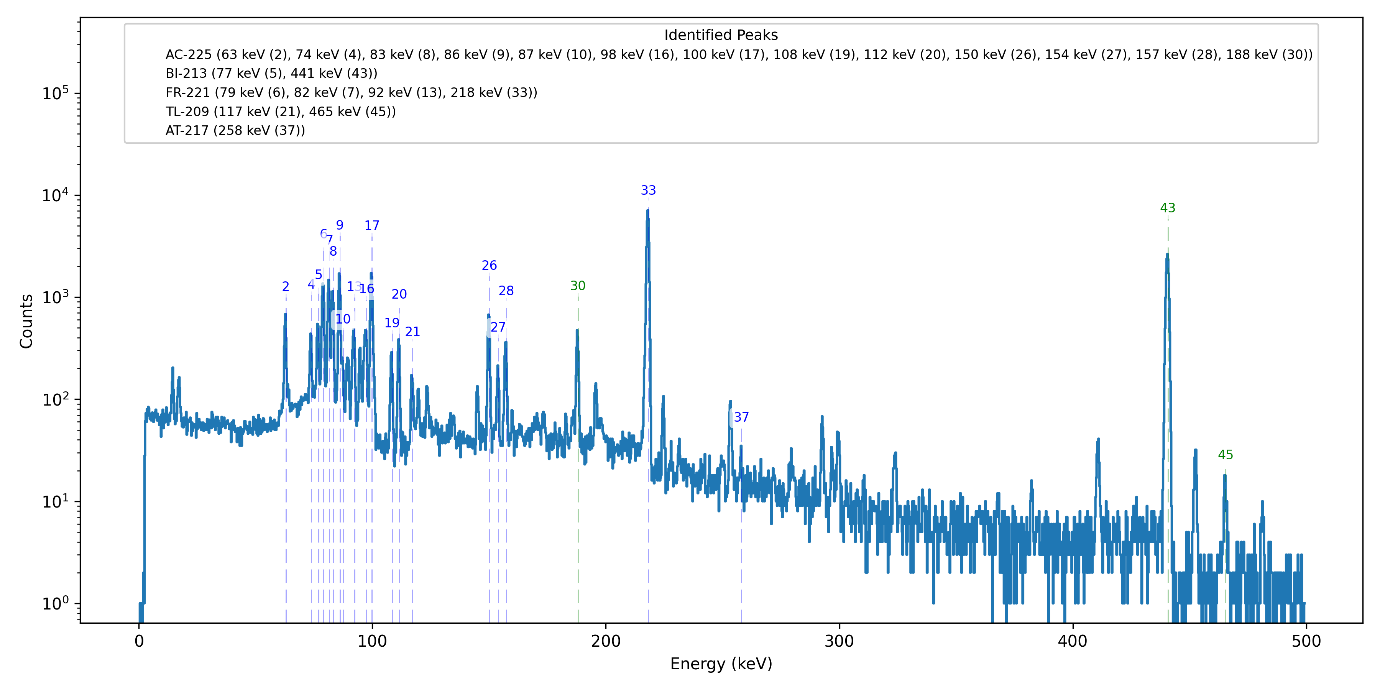


**Biodistribution of ^221^Fr in mice injected with ^221^Fr**


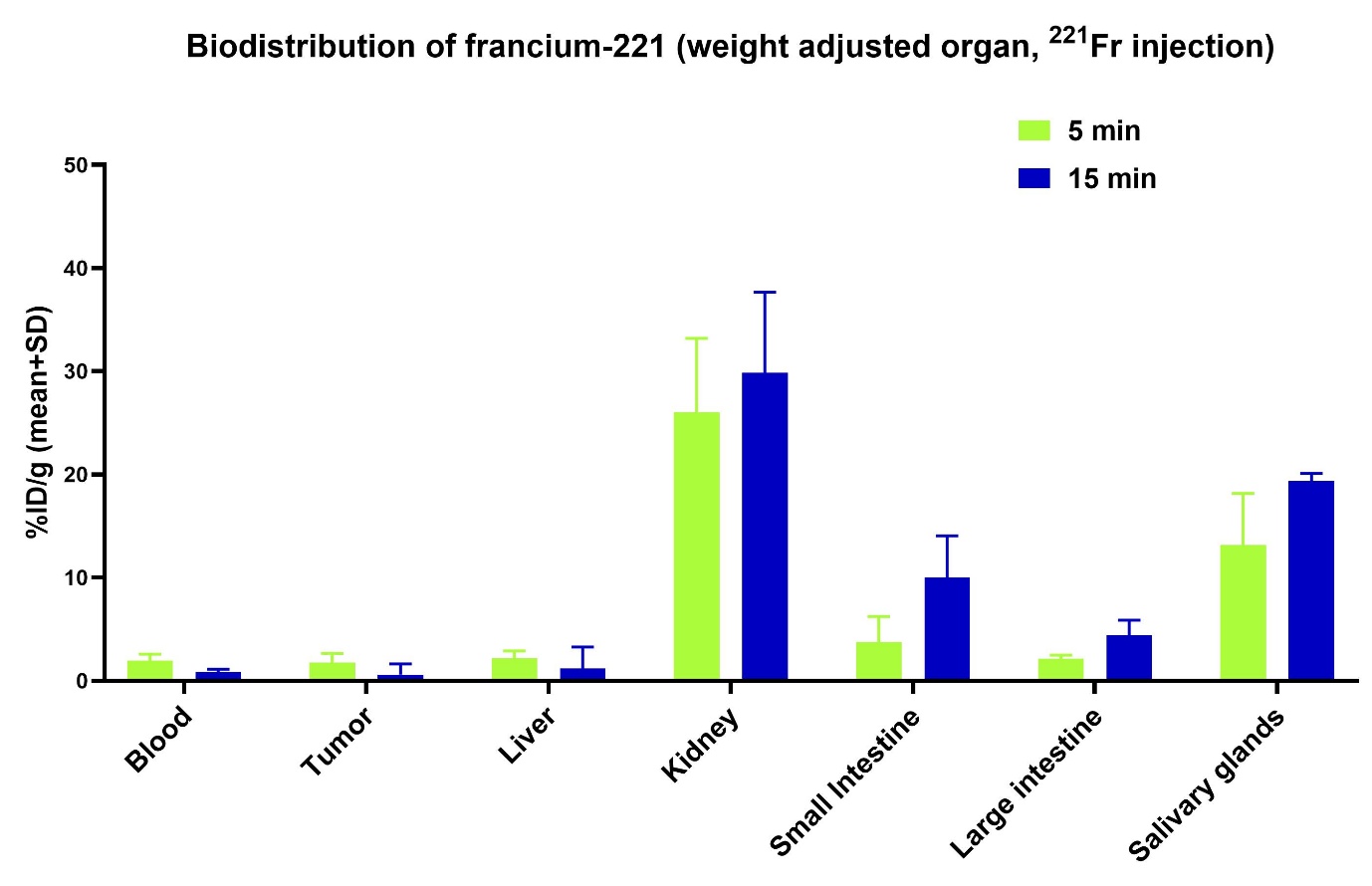


**Figure S1**: Biodistribution data from injection of ^221^Fr in LNCaP tumor bearing mice. Results obtained from measuring with a HPGe detector depicted in percentage of injected dose per gram organ. The values are mean values with standard deviation (n=3 per time point) considering linear propagation of uncertainties.

**Biodistribution of ^213^Bi in mice injected with ^213^Bi**


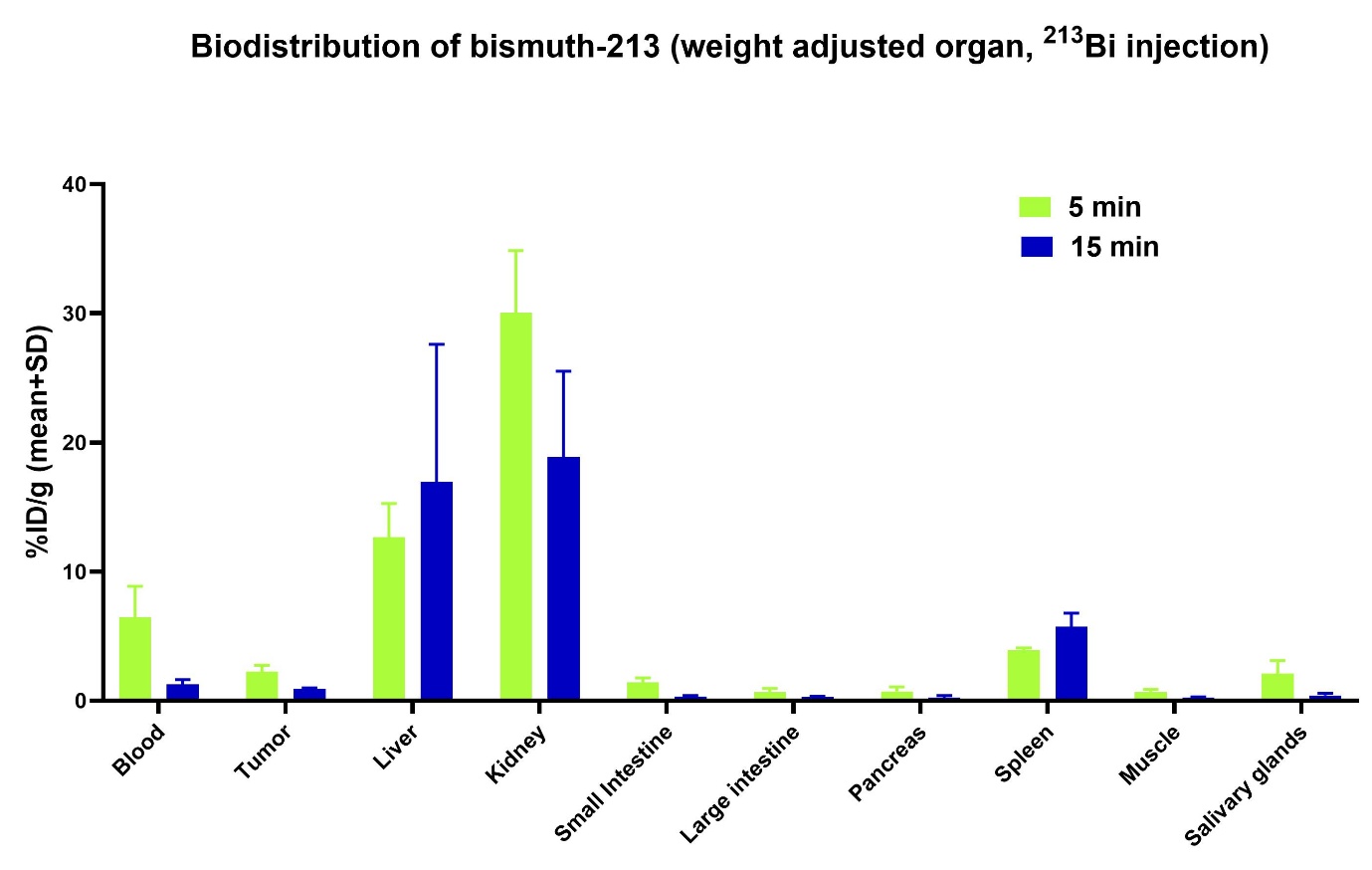


**Figure S2**: Biodistribution data from injection of ^213^Bi in LNCaP tumor bearing mice. Results obtained from measuring with a HPGe detector depicted in percentage of injected dose per gram organ. The values are mean values with standard deviation (n=3 per time point) considering linear propagation of uncertainties.
